# Expression of phenylalanine ammonia lyases in *Synechocystis* sp. PCC 6803 and subsequent improvements of sustainable production of phenylpropanoids

**DOI:** 10.1186/s12934-021-01735-8

**Published:** 2022-01-10

**Authors:** Kateryna Kukil, Pia Lindberg

**Affiliations:** Microbial Chemistry, Department of Chemistry - Ångström, Uppsala University, Box 523, SE 751 20 Uppsala, Sweden

**Keywords:** *Synechocystis* sp*.* PCC 6803, Phenylalanine ammonia lyase, *trans*-cinnamic acid, *p-*coumaric acid, Shikimate pathway, Laccase, High-density cultivation

## Abstract

**Background:**

Phenylpropanoids represent a diverse class of industrially important secondary metabolites, synthesized in plants from phenylalanine and tyrosine. Cyanobacteria have a great potential for sustainable production of phenylpropanoids directly from CO_2_, due to their photosynthetic lifestyle with a fast growth compared to plants and the ease of generating genetically engineered strains. This study focuses on photosynthetic production of the starting compounds of the phenylpropanoid pathway, *trans*-cinnamic acid and *p-*coumaric acid, in the unicellular cyanobacterium *Synechocystis* sp. PCC 6803 (*Synechocystis*).

**Results:**

A selected set of phenylalanine ammonia lyase (PAL) enzymes from different organisms was overexpressed in *Synechocystis*, and the productivities of the resulting strains compared. To further improve the titer of target compounds, we evaluated the use of stronger expression cassettes for increasing PAL protein levels, as well as knock-out of the laccase gene *slr1573*, as this was previously reported to prevent degradation of the target compounds in the cell. Finally, to investigate the effect of growth conditions on the production of *trans*-cinnamic and *p-*coumaric acids from *Synechocystis*, cultivation conditions promoting rapid, high density growth were tested. Comparing the different PALs, the highest specific titer was achieved for the strain AtC, expressing PAL from *Arabidopsis thaliana*. A subsequent increase of protein level did not improve the productivity. Production of target compounds in strains where the *slr1573* laccase had been knocked out was found to be lower compared to strains with wild type background, and the Δ*slr1573* strains exhibited a strong phenotype of slower growth rate and lower pigment content. Application of a high-density cultivation system for the growth of production strains allowed reaching the highest total titers of *trans*-cinnamic and *p-*coumaric acids reported so far, at around 0.8 and 0.4 g L^−1^, respectively, after 4 days.

**Conclusions:**

Production of *trans*-cinnamic acid, unlike that of *p-*coumaric acid, is not limited by the protein level of heterologously expressed PAL in *Synechocystis*. High density cultivation led to higher titres of both products, while knocking out *slr1573* did not have a positive effect on production. This work contributes to capability of exploiting the primary metabolism of cyanobacteria for sustainable production of plant phenylpropanoids.

**Supplementary Information:**

The online version contains supplementary material available at 10.1186/s12934-021-01735-8.

## Background

Aromatic amino acids (AAA) and their derivatives phenylpropanoids, represent a large group of plant secondary metabolites that includes flavonoids, coumarins, stilbensenes, lignols, and catechin. These compounds are widely applicable as food additives, fragrances, cosmetics, nutraceuticals, and for production of antibacterial, antitumor, antiviral drugs and other pharmaceuticals [1–4]. The huge market demand for plant bioactive secondary metabolites is at present mostly fulfilled through demanding and inefficient extraction from plant tissues, or through chemical synthesis based on fossil resources, which in many cases is difficult to achieve due to the structural complexity of the desired metabolites and often results in mixtures of isomers. Microbial synthesis of such compounds presents an attractive alternative. Engineered microorganisms can be designed to generate more of a desired product per cell than what can be achieved in plants, while at the same time, the biosynthetic pathways in the host microorganisms can be very specific to generate only one product, and the production will be scalable and more sustainable [5–7].

**Fig. 1:**
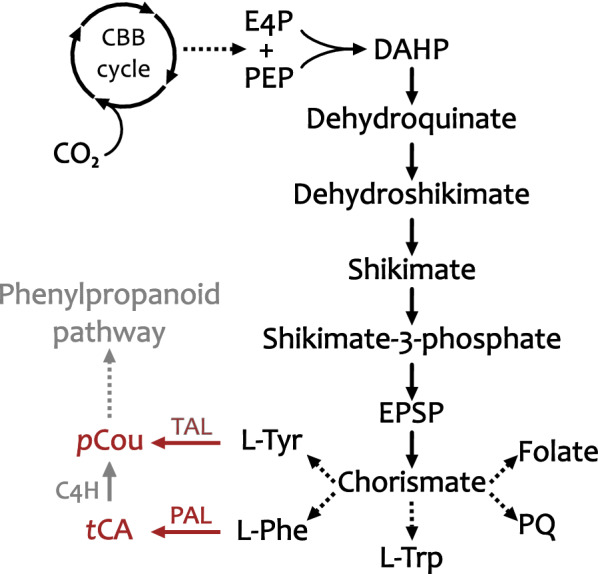
Schematic overview of Shikimate pathway for biosynthesis of aromatic amino acids in *Synechocystis*. C4H, cinnamate-4-hydroxylase; CBB cycle, Calvin-Benson-Bassham cycle; DAHP, 3-deoxy-d-arabinoheptulosonate 7-phosphate synthase; E4P, erythrose-4-phosphate; EPSP, 5-enolpyruvylshikimate-3-phosphate synthase; l-Phe, l-phenylalanine; l-Trp, l-tryptophane; l-Tyr, l-tyrosine; PAL, phenylalanine ammonia lyase; *p*Cou, *p-*coumaric acid; PEP, phosphoenolpyruvate; PQ, plastoquinone; TAL, tyrosine ammonia lyase; *t*CA, *trans*-cinnamic acid

Engineered cyanobacteria exhibit a great potential for sustainable production of plant phenylpropanoids by direct conversion of CO_2_. Just as other autotrophs, cyanobacteria possess a pathway for de novo biosynthesis of AAA phenylalanine (Phe), tyrosine (Tyr) and tryptophan (Trp) from the common precursor chorismate through the shikimate pathway (Fig. 1) (Kyoto Encyclopedia of Genes and Genomes (KEGG); https://www.genome.jp/kegg/). The pathway starts from the condensation of erythrose-4-phosphate (E4P) and phosphoenolpyruvate (PEP), derived from CO_2_ fixation in central carbon metabolism, to form the first compound in the shikimate pathway, 3-deoxy-d-arabinoheptulosonate 7-phosphate synthase (DAHP). This first step is catalyzed by the enzyme DAHP synthase. This enzyme in microorganisms, supposedly including cyanobacteria, is subjected to tight regulation by feedback inhibition by the products of the pathway [8, 9]. Through six consecutive reactions, DAHP undergoes cyclisation to form chorismate, which in cyanobacteria is a common substrate for the three AAA, plastoquinone, and folic acid [10–12]. From chorismate, by one additional step, Phe can be non-oxidatively deaminated to form *trans*-cinnamic acid (*t*CA) by the action of an enzyme called phenylalanine ammonia lyase (PAL, EC 4.3.1.24). The PAL enzymatic reaction plays a key role in synthesis of phenylpropanoids, linking secondary metabolites synthesis with primary metabolism. In plants, *t*CA can be further converted to *p*-coumaric acid (*p*Cou, or otherwise named hydroxycinnamic acid) by membrane bound cinnamate-4-hydroxylase (C4H) [13]. Alternatively to C4H, the enzyme tyrosine ammonia lyase (TAL, EC 4.3.1.25) which was discovered in bacteria, catalyzes a non-oxidative deamination to form *p*Cou directly from Tyr. PAL and TAL are widely spread in nature, however in bacteria their role is connected to synthesis of the chromophore of photoactive yellow protein and antibiotics [14–16] rather than synthesis of flavonoids and coumarins as in plants [17]. PAL was also discovered in filamentous cyanobacteria, but not in unicellular strains, and yet its role in metabolism in these organisms has not been elucidated [18].

A distinct class of PALs is represented by bifunctional enzymes that can utilize both Phe and Tyr with close to similar efficiencies [19, 20]. The ability of an enzyme to use both substrates led to investigation of a possible region in the protein amino acid sequence that might be responsible for substrate preference. The discovery of such a region, called substrate selectivity switch, showed that a single amino acid substitution can dictate whether an enzyme will use Phe or Tyr as a substrate [21]. PAL and TAL together with histidine ammonia lyase (HAL, EC:4.3.1.3) belong to the ammonia lyase family of proteins. All three of them share the common characteristic of a modified prosthetic group, 3,5-dihydro-5-methylidine-4H-imidazol-4-one (MIO), a unique prosthetic group which is formed autocatalytically from a highly conserved Ala-Ser-Gly tripeptide sequence (ASG motif) [22]. Crystal structure studies revealed that eukaryotic PALs differ from prokaryotic being ~ 20 kDa larger by the presence of N-terminus extension and an additional insertion domain. This additional domain forms an arch-like structure over the active site, that proposedly is acting as a shielding domain which restricts substrate entry to the active site, however its exact role is unknown [18, 23, 24].

Cyanobacterial AAA biosynthesis is less studied than that in plants, yeasts and some other bacterial hosts like *Escherichia coli*, presenting a challenge for engineering. Nevertheless, the photosynthetic nature of cyanobacteria and their faster growth rate than that of plants make them good candidates for sustainable production of AAA and phenylpropanoids, and a few reports have described cyanobacteria engineered for this purpose. To date, cyanobacterial production of *t*CA, caffeic acid [25], *p*Cou [26–28], and phenylethanol [29] has been demonstrated. The common strategies implemented to improve the productivity in cyanobacteria were enhancing the carbon flux into shikimate pathway by reliving the feedback inhibition of key steps in the pathway, such as overexpression of feedback-resistant DAHP synthase and feedback-resistant chorismate mutase/prephenate dehydratase from *E. coli*. Xue and coworkers [26] discovered a putative laccase in *Synechocystis* sp. PCC 6803 encoded by *slr1573*, and deletion of this gene led to increased *p*Cou titers, presumably due to involvement of the laccase in oxidation of phenolic compounds. Another strategy for productivity boost consisted in knocking-out of a competing pathway: deletion of arogenate dehydrogenase encoded by *tyrA* from the terminal branch of Tyr biosynthesis led to the enhancement of the two-step *p*Cou biosynthesis pathway via Phe and *t*CA. However, it is not clear how the cells are able to survive a knockout of this supposed essential gene, unless other reactions leading to Tyr synthesis exist in cyanobacteria [30].

In this study we comparatively express and test several PALs originating from different organisms for the production of *t*CA in the model cyanobacterium *Synechocystis* sp. PCC 6803 (hereafter *Synechocystis*), in strains with and without the *slr1573* laccase gene. We also test engineered strains under more optimal growth conditions; recently, a high-density cultivation system exhibited a great potential of reaching high cumulative titer of desired product in short period of time [31–33]. The reaction performed by PAL is of industrial interest since it opens a gateway to generating a myriad of highly valuable secondary plant metabolites, non-native to cyanobacteria*.*

## Results and discussion

### Comparative production of *t*CA

In this study we aimed to use several PAL enzymes in order to test and select the one most favorable for *t*CA production in *Synechocystis*. For this, we searched the literature for available kinetic data on purified PAL proteins, and from available data choose a set of genes with different origins and characteristics. Selected candidates for *pal* genes were from two plant species, *Petroselinum crispum* (*Pc*) [34] and *Arabidopsis thaliana* (*At*) [35], and two filamentous cyanobacteria *Nostoc punctiforme* (*Np*), *Anabaena variabilis* (*Av*) [18]. For these PAL candidates, in vitro studies on their biochemical properties were available in the literature. In addition to these already purified and described PAL enzymes, we also decided to include a fifth PAL candidate, based on the PAL evolutionary study of Hemmati [36]. In that study, a phylogenic tree generated for 369 aligned amino acid sequences of PAL showed that several PAL enzymes do not cluster together. As suggested by the author, those PAL enzymes are probably encoded by *pal* isozymes with unique functional genes. Among several options from such unclustered PAL candidates, our choice fell to the bacterial PAL from *Treponema socranskii subsp. paredis* ATCC 35535 (*Ts*). The choice of this PAL candidate was partially based on the unusual amino acids residues at the positions 86 and 87. These residues, which correspond to the substrate specificity [21], are phenylalanine and histidine in the case of *Ts* PAL instead of the more canonical pair phenylalanine-leucine for PAL or histidine-leucine for TAL properties.

**Fig. 2:**
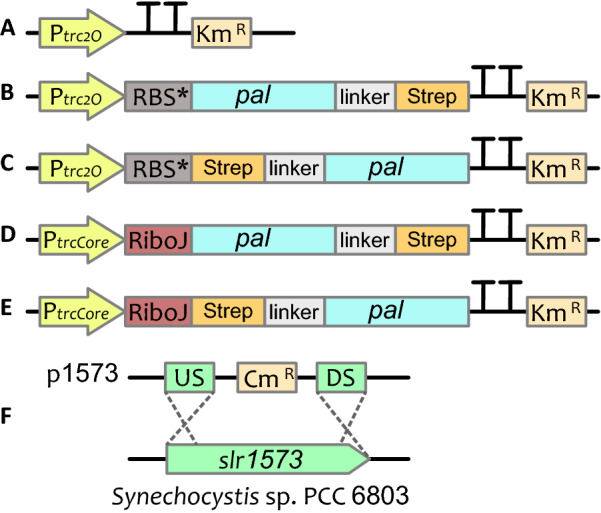
Schematic representation of genetic constructs used in this work. **A** an illustration of pEEK* control construct, **B** pEEK1, **C** pEEKN, **D** pEEK3C, **E** pEEK3, **F** illustration of integrative plasmid p1573, where US and DS are upstream and downstream regions for homologous recombination for knock-out of *slr1573* gene in *Synechocystis*

The five genes encoding different PAL candidates were cloned in two types of expression vectors (Fig. 2): pEEK [37], and its derivative pEEKN, and successfully conjugated into *Synechocystis* (see Table 1 for a list of plasmid backbones used and strains created in this study)*.* In this approach, each *pal* gene is expressed in two versions, with either an N- or a C-terminal Strep-tag in order to evaluate the protein expression and production of *t*CA in *Synechocystis*. An empty vector control strain was obtained by conjugating *Synechocystis* with the pEEK* plasmid, which only contains the Km antibiotic resistance.

**Table 1:** List of plasmid backbones and *Synechocystis* strains used in this study

Construct	Comment	Antibiotics resistance	References
pEEK	RSF 1010-based expression vector with *ccdB* flanked by cloning sites and the ribosome binding site RBS*, promoter P*trc2O*, and a C-terminal Strep-tag sequence	Km	[37]
pEEK*	Same as pEEK but without *ccdB*, used as empty vector control	Km	Englund, E., not published
pEEKN	Same as pEEK but with N-terminal Strep-tag sequence	Km	This study
pEEK3	Based on pEEC3 [38], with P*trcCore* promoter, RiboJ and N-terminal Strep-tag sequence, but with Km resistance	Km	Englund, E., not published
pEEK3C	Same as pEEK3 but with C-terminal Strep-tag sequence	Km	This study
pEERM3	Empty backbone based on pJ344 vector backbone for integration into neutral site 1 (*slr1068*) of *Synechocystis* 6803, carrying P*nrsB* promotor and RBS*	Cm	[39]
p1573	Same as pEERM3 but with homologous recombination regions to *slr1573*	Cm	This study

Strains	Comment	Antibiotics resistance	References
EvC	Control strain of *Synechocystis* WT harboring pEEK* plasmid for Km resistance	Km	This study
PcC	*Petroselinum crispum* PAL expressed from P*trc* RBS* in pEEK	Km	This study
AtC	*Arabidopsis thaliana* PAL expressed from P*trc* RBS* in pEEK	Km	This study
NpC	*Nostoc punctiforme* PAL expressed from P*trc* RBS* in pEEK	Km	This study
AvC	*Anabaena variabilis* PAL expressed from P*trc* RBS* in pEEK	Km	This study
TsC	*Treponema socranskii* PAL expressed from P*trc* RBS* in pEEK	Km	This study
TsC-H87L	*Treponema socranskii* PAL with mutation H87L expressed from P*trc* RBS* in pEEK	Km	This study
PcN	*Petroselinum crispum* PAL expressed from P*trc* RBS* in pEEKN	Km	This study
AtN	*Arabidopsis thaliana* PAL expressed from P*trc* RBS* in pEEKN	Km	This study
NpN	*Nostoc punctiforme* PAL expressed from P*trc* RBS* in pEEKN	Km	This study
AvN	*Anabaena variabilis* PAL expressed from P*trc* RBS* in pEEKN	Km	This study
TsN	*Treponema socranskii* PAL expressed from P*trc* RBS* in pEEKN	Km	This study
TsN-H87L	*Treponema socranskii* PAL with mutation H87L expressed from P*trc* RBS* in pEEKN	Km	This study
Pc3C	*Petroselinum crispum* PAL expressed from P*trc* RiboJ in pEEK3C	Km	This study
At3C	*Arabidopsis thaliana* PAL expressed from P*trc* RiboJ in pEEK3C	Km	This study
Np3C	*Nostoc punctiforme* PAL expressed from P*trc* RiboJ in pEEK3C	Km	This study
Av3C	*Anabaena variabilis* PAL expressed from P*trc* RiboJ in pEEK3C	Km	This study
Ts3C	*Treponema socranskii* PAL expressed from P*trc* RiboJ in pEEK3C	Km	This study
Ts3C-H87L	*Treponema socranskii* PAL with mutation H87L expressed from P*trc* RiboJ in pEEK3C	Km	This study
Pc3N	*Petroselinum crispum* PAL expressed from P*trc* RiboJ in pEEK3	Km	This study
At3N	*Arabidopsis thaliana* PAL expressed from P*trc* RiboJ in pEEK3	Km	This study
Np3N	*Nostoc punctiforme* PAL expressed from P*trc* RiboJ in pEEK3	Km	This study
Av3N	*Anabaena variabilis* PAL expressed from P*trc* RiboJ in pEEK3	Km	This study
Ts3N	*Treponema socranskii* PAL expressed from P*trc* RiboJ in pEEK3	Km	This study
Ts3N-H87L	*Treponema socranskii* PAL with mutation H87L expressed from P*trc* RiboJ in pEEK3	Km	This study
EvC*Δslr157*3	Control strain of *Synechocystis* WT harboring pEEK* plasmid for Km resistance with knocked-out *slr1573*	Km, Cm	This study
AtC*Δslr1573*	*Arabidopsis thaliana* PAL expressed from P*trc* RBS* in pEEK with knocked-out *slr1573*	Km, Cm	This study
AtN*Δslr1573*	*Arabidopsis thaliana* PAL expressed from P*trc* RBS* in pEEKN with knocked-out *slr1573*	Km, Cm	This study
TsCΔ*slr1573*	*Treponema socranskii* PAL expressed from P*trc* RBS* in pEEK with knocked-out *slr1573*	Km, Cm	This study
TsN*Δslr1573*	*Treponema socranskii* PAL expressed from P*trc* RBS* in pEEKN with knocked-out *slr1573*	Km, Cm	This study

Interestingly, when initially all of the *pal* expression constructs were tested in *E. coli* for the corresponding *t*CA or *p*Cou presence in the culture medium, it was revealed that *Ts pal* encodes a Tyrosine ammonia lyase, producing *p*Cou from Tyr (see Additional file 1: Fig. S1). To explore a possibility that by switching the histidine at position 87 to leucine, the substrate specificity would change from Tyr to Phe, we performed site directed mutagenesis on the *Ts tal* sequence. As a result, it was found that the mutation *Ts*–H87L successfully altered the enzyme selectivity towards phenylalanine, as no *p*Cou was accumulating in the medium (Additional file 1: Fig. S1). The *Ts*-H87L *pal* was therefore included in the study, and was cloned and conjugated into *Synechocystis* the same way as the other *pal* CDS.

**Fig. 3:**
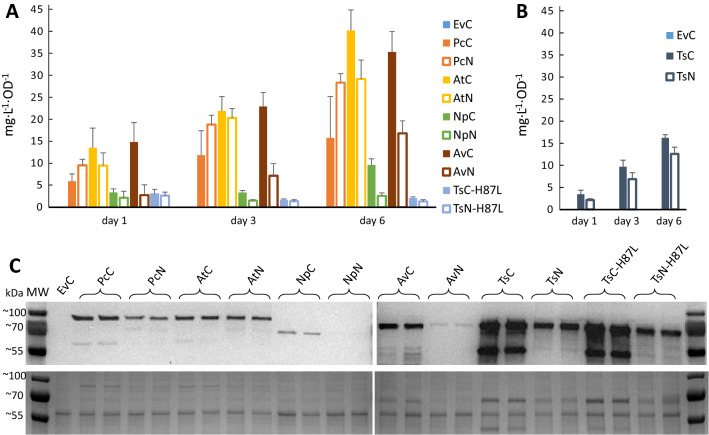
Productivity and Western Blot of *Synechocystis* strains overexpressing PAL or TAL from the pEEK vector. **A** *t*CA production in strains EvC, PcC, PcN, AtC, AtN, NpC, NpN, AvC, AvN, TsC-H87L and TsN-H87L. **B** *p*Cou production in strains TsC and TsN. Samples of growth medium were taken on day one, three and six. The values are the means of three biological replicates and two technical replicates, error bars represent the standard deviation. **A** and **B** are plotted using data from Table 2. **C** Western Blot and SDS-PAGE of extracts from engineered *Synechocystis* strains (EvC, PcC, PcN, AtC, AtN, NpC, NpN, AvC, AvN, TsC-H87L, TsN-H87L, TsC and TsN) analyzed for presence of PAL proteins. Upper panel displays the Western Blot using anti-Strep antibody; bottom panel shows the SDS-PAGE loaded with 5 µg of soluble fraction from protein crude extract of the different strains

**Fig. 4:**
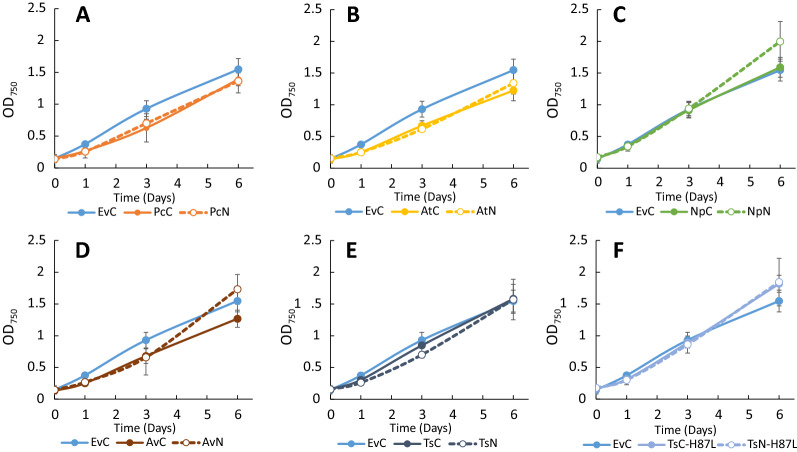
Growth of *Synechocystis* strains overexpressing PAL or TAL from the pEEK during 6 days experiment. **A** Strains EvC, PcC, PcN; **B** strains EvC AtC, AtN; **C** strains EvC, NpC, NpN; **D** strains EvC, AvC, AvN; **E** strains EvC, TsC TsN; **F** strains EvC, Ts3C-H87L and Ts3N-H87L. Results are the mean of three biological replicates, error bars represent standard deviation

Cultures of the engineered *Synechocystis* strains were grown for six days under constant light, and samples for OD_750_ and LC–MS analysis for *t*CA and *p*Cou content in the culture supernatant were taken on days one, three and six (Fig. 3A and B, Table 2). The highest specific production of *t*CA was observed by strain AtC, reaching the value of 40.2 ± 5.6 mg L^−1^ OD^−1^ of *t*CA at day six (Fig. 3A, Table 2). Western Blot analysis of proteins in the engineered strains at the last day of the experiment (Fig. 3C) showed that expression levels of C-terminally tagged PAL proteins were higher than N-terminally tagged, which correlates with higher titers of the product in most of the cases. The extra bands of smaller molecular size than the PALs may be due to degradation of the detected proteins. The cause of the observed differences in expression levels of the same PAL dependent on the tag position is uncertain. Unpredictability of expression levels is often caused by different 5 UTR′ sequences, resulting from combination of promoters and RBSs, which together with different coding sequences can create secondary structures restricting efficient ribosome binding [40, 41]. The N-terminal Strep-tag sequence may be expected to even out such context-dependent differences, however, our results showed weaker and more uneven protein expression levels for strains with N-terminally tagged PALs. Another possible explanation is a lower stability of PAL proteins when expressed with N-terminal tag, or that the N-terminal tag sequence affects mRNA stability. The differences in production levels of target compounds might also be by virtue of differences in enzyme activity of expressed PALs due to the fused Strep-tag peptide, as the AtN and AtC strains demonstrated similar protein levels but different production of *t*CA per cell (Fig. 3A and C). The growth of engineered strains which produced more of the molecule of interest per cell over the time of experiment (Fig. 4) was slower compared to the EvC. This result indicates that the expression of PAL protein, which consumes the central metabolite Phe, negatively affects the growth.

**Table 2:** *t*CA and *p*Cou production by engineered *Synechocystis* strains after six days of photoautotrophic growth

Strain	mg L^−1^ OD_750_^−1^	mg L^−1^	Strain	mg L^−1^ OD_750_^−1^	mg L^−1^
***t*CA production**					
EvC	N/D				
PcC	15.8 ± 9.4	26.3 ± 13.4	Pc3C	20.7 ± 3.3	27.5 ± 4.7
PcN	28.3 ± 2	37.3 ± 6.1	Pc3N	32.9 ± 3.2	46.1 ± 7.6
AtC	40.2 ± 5.6	49.6 ± 8.7	At3C	32.5 ± 1.6	41.0 ± 4.9
AtN	29.1 ± 4.3	35.8 ± 3	At3N	32.6 ± 3.6	45.5 ± 3.2
NpC	9.6 ± 1.4	14.1 ± 1.6	Np3C	32.7 ± 5.4	38.0 ± 7.1
NpN	2.6 ± 0.7	4.8 ± 1.4	Np3N	22.8 ± 8.5	29.1 ± 10.6
AvC	35.3 ± 4.6	46.3 ± 6.6	Av3C	29.5 ± 5.1	29.3 ± 7
AvN	16.8 ± 2.9	26.7 ± 5.5	Av3N	30.7 ± 9.7	30.2 ± 13.7
TsC-H87L	0.3 ± 0.3	3.8 ± 0.7	Ts3C-H87L	4 ± 0.4	4.7 ± 0.7
TsN-H87L	0.2 ± 0.3	2.5 ± 0.7	Ts3N-H87L	2.7 ± 0.2	3.9 ± 0.7
TsC	Trace amounts		Ts3C	Trace amounts	
TsN	Trace amounts		Ts3N	Trace amounts	
***p*Cou production**					
TsC	16.2 ± 0.7	25.6 ± 3.9	Ts3C	23.5 ± 1.2	22.4 ± 3.2
TsN	12.6 ± 1.5	19.2 ± 5.6	Ts3N	22.7 ± 2.4	28.3 ± 3.9
TsC-H87L	N/D		Ts3C-H87L	N/D	
TsN-H87L	N/D		Ts3N-H87L	N/D	

**Fig. 5:**
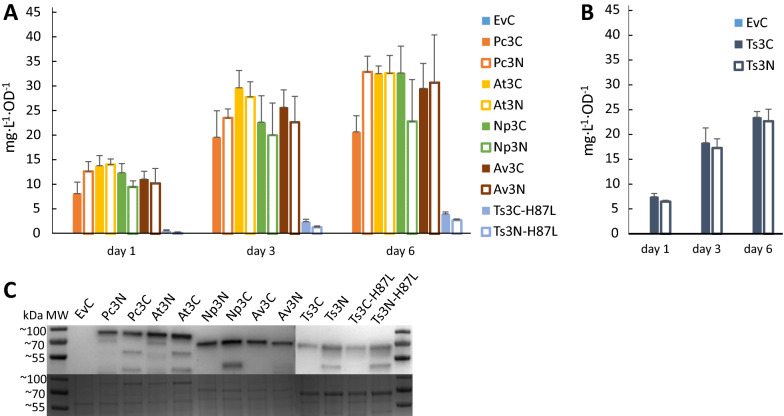
Productivity and Western Blot of *Synechocystis* strains overexpressing PAL or TAL from the pEEK3 vector. **A** Comparison of *t*CA production in engineered *Synechocystis* strains EvC, Pc3C, Pc3N, At3C, At3N, Np3C, Np3N, Av3C, Av3N, Ts3C-H87L and Ts3N-H87L. **B** *p*Cou production in strains Ts3C and Ts3N. Samples of growth medium were taken on day one, three and six. The values are the means of three biological replicates and two technical replicates, error bars represent the standard deviation. **A** and **B** are plotted using the data from Table 2. **C** Western Blot and SDS-PAGE of extracts from engineered *Synechocystis* strains (EvC, Pc3C, Pc3N, At3C, At3N, Np3C, Np3N, Av3C, Av3N, Ts3C, Ts3N, Ts3C-H87L and Ts3N-H87L) analyzed for presence of PAL proteins. Upper panel displays the Western Blot using anti-Strep antibody; bottom panel shows the SDS-PAGE loaded with 3 µg of soluble fraction from protein crude extract of the different strains

Data corresponds to the panels A and B in Figs. 3 and 5

N/D, none detected

The production of *p*Cou by strains TsC and TsN showed similar values, although TsC protein levels were significantly higher than those of the TsN strain (Fig. 3B, Table 2). Substitution of one amino acid residue, H87L, in Ts TAL had successfully switched the enzyme’s substrate selectivity from Tyr to Phe. However, the activity of the mutated enzyme variant was decreased compared to a wild type variant, as the productivity per cell decreased nearly eight times, whereas the protein expression levels remained similar (Fig. 3).

### Enhancement of *t*CA production via increase of protein expression level

Since the productivity in our first experiments seemed to correlate with protein expression levels, to further improve *t*CA production in *Synechocystis* we intended to increase protein expression levels of PALs. For this, the *pal* genes were cloned into two vectors, pEEK3 and pEEK3C, and successfully conjugated into *Synechocystis*. In pEEK3, the expression is driven by a strong constitutive promotor P*trcCore*, followed by a sequence encoding the self-cleaving ribozyme RiboJ [38, 42]. RiboJ causes the transcribed mRNA to undergo cleaving of its own 5′UTR, which facilitates biding of the ribosome to the RBS of the transcript. Application of RiboJ has been reported to enable a strong, predictable expression in *Synechocystis* [38, 43].

**Fig. 6:**
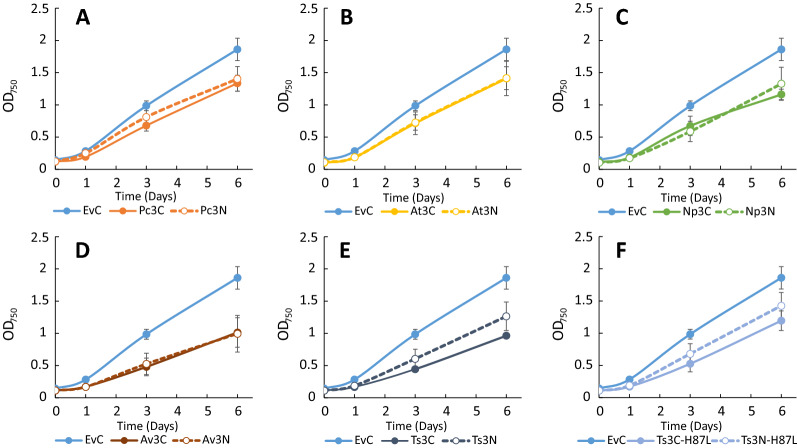
Growth of *Synechocystis* strains overexpressing PAL or TAL from the pEEK3 during 6 days experiment. **A** strains EvC, Pc3C, Pc3N; **B** strains EvC At3C, At3N; **C** strains EvC, Np3C, Np3N; **D** strains EvC, Av3C, Av3N; **E** strains EvC, Ts3C Ts3N; **F** strains EvC, Ts3C-H87L and Ts3N-H87L. Results are the mean of three biological replicates, error bars represent standard deviation

**Fig. 7:**
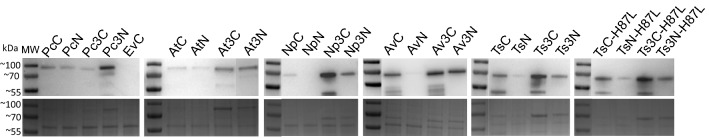
PAL and TAL relative expression in cell extracts at day six of comparative growth experiments. Upper panel displays the Western Blot using anti-Strep antibody; bottom panel shows the SDS-PAGE loaded with 5 µg of soluble fraction from protein crude extract of the different strains, except for strains TsC, TsN, Ts3C, Ts3N, TsC-H87L, TsN-H87L, Ts3C-H87L and Ts3N-H87L where 2 µg of protein was loaded

Comparative growth experiment on these strains demonstrated a similar range of *t*CA production per cell among all strains, except for the Ts3-H87L strains (Fig. 5A, Table 2). A normalized maximal titer of approx. 32 mg L^−1^ OD^−1^ was reached by several strains, namely Pc3N, At3C, At3N, Np3C (Fig. 5A, Table 2). The *p*Cou specific production titer was increased for Ts3C and Ts3N strains as compared to TsC and TsN strains (Fig. 5B, Table 2). In addition, Western Blot analysis (Fig. 5C) also showed similar expression levels of different PALs with lesser variation dependent on the Strep-tag position. Compared to engineered strains based on overexpression in pEEK, the growth of all engineered strains based on overexpression in pEEK3 showed a larger growth impairment as compared to EvC (Fig. 6). However, the obtained productivity values were lower than the titer achieved for the AtC strain. These findings prompted us to perform a comparison of the protein expression level for each PAL in all engineered strains. Figure 7 shows the comparative expression of each PAL overexpression variant and we can observe that for all pEEK3 based strains, expression was higher than for corresponding pEEK based strains. The difference in the relative expression levels of the same pEEK3 strains in Figs. 5C, 7 might be due to variation between protein extracts from different biological replicates of same strain. Nonetheless, we can conclude that more PAL protein per cell did not lead to higher *t*CA productivity, and the usage of RiboJ helped to decrease the differences between C- and N-terminal Strep-tag position in expression constructs. These results indicate that the PAL expression level does not seem to be the bottleneck of *t*CA production in *Synechocystis*. This is further supported by the fact that although all PALs used in this study possess different kinetic parameters as was shown on purified proteins [18, 34, 35], their overexpression with similar protein levels in *Synechocystis* resulted in nearly similar production titers of product *t*CA. The apparent limitation once a high enough protein level has been reached might thus be in the intracellular levels of the substrate phenylalanine and the metabolic regulation of its biosynthesis. The obtained production of 40.2 ± 5.6 mg L^−1^ OD_750_^−1^ of *t*CA might represent an experimental maximum under the tested conditions, corresponding to the “limit” of phenylalanine consumption before causing detrimental effect on cell fitness.

### Effect of laccase knockout on *t*CA production

The discovery by Xue et al. [26] of a laccase enzyme encoded by gene *slr1573* in *Synechocystis* was related to the role of this enzyme in the decomposition of *p*Cou inside the cell. When the laccase gene was knocked out, the authors detected a 25-fold increase in *p*Cou titer. To assess if the *t*CA can be decomposed in our engineered strains by the action of the laccase, we constructed an integration plasmid, p1573 (Fig. 2F, Table 1), to knock out this gene in the most prominent producing strains in our study—AtC and AtN. TsC and TsN strains were also used as the control strains of *p*Cou production. The EvC was transformed with the p1573 plasmid to form strain EvCΔ*slr1573,* serving as a growth control strain (Table 1).

**Fig. 8:**
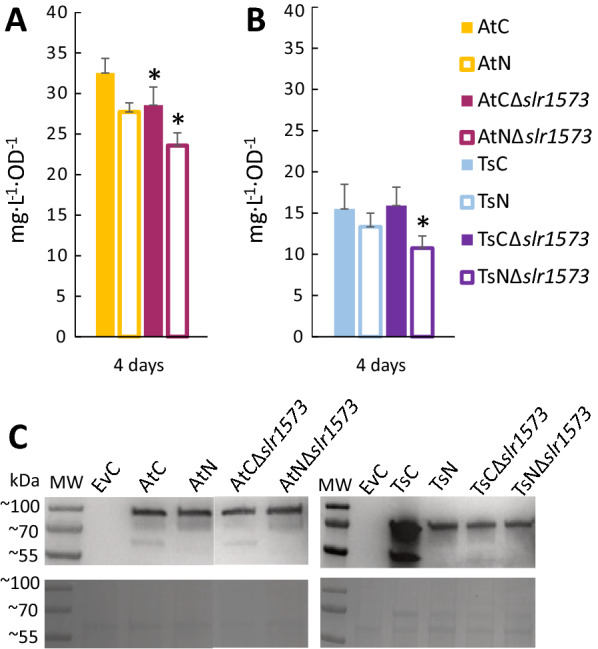
Comparisons of *Δslr1573* and WT background strains. **A** Comparison of *t*CA production in engineered *Synechocystis* strains Atc, AtN, AtCΔ*slr1573* and AtN*Δslr1573*; **B** comparison of *p*Cou production in strains TsC, TsN, TsCΔ*slr1573* and TsN*Δslr1573*. Samples of growth medium were taken on day four. The values are the means of three biological replicates and two technical replicates, error bars represent the standard deviation. **C** Western Blot and SDS-PAGE of cell extracts at day four of comparative growth experiments analyzed for presence of PAL proteins. Upper panel displays the Western Blot using anti-Strep antibody; bottom panel shows the SDS-PAGE loaded with 5 µg of soluble fraction from protein crude extract of the different strains

**Fig. 9:**
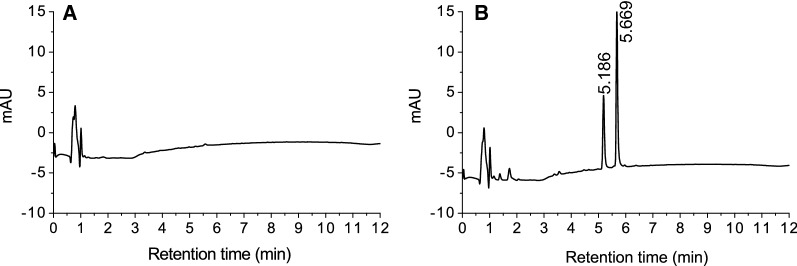
LC–MS profile of culture medium at 275 nm wavelength on day four from comparative experiment. **A** profile from EvC strain; **B** profile from EvC*Δslr1573* strain

Comparative growth experiments with the original and laccase knockout strains were performed during four days under constant moderate light intensity of 45 µmol photons m^−2^ s^−1^. The productivity on the last days of experiment showed lower *t*CA and *p*Cou titers for the Δ*slr1573* strains (Fig. 8). These results are different from what was observed before by Xue et al. [26], where knocking out *slr1573* had a strong positive effect on *p*Cou titers. Moreover, on the LC–MS profile of *p*Cou production by TsC and TsN strains we did not detect additional peaks that may correspond to the polymerization of 4-vinylphenol as observed by Xue et al. [26]. This difference may however depend on different extraction procedures of *p*Cou: extraction of media with ethyl acetate [26, 27] whereas in our study we subjected the supernatant from the culture directly to LC–MS analysis. With organic solvents extraction in those studies it was possible to extract polymeric compounds with low water solubility. Furthermore, we observed two unidentified peaks of high molecular weight (m/z 323) (see Additional file 1: Fig. S2) in the LC–MS profile from all Δ*slr1573* strains including the control strain EvCΔ*slr1573* (Fig. 9). These peaks may correspond to the accumulation of phenolic compounds or polymers due to the disruption of their native pathway in the cell.

**Fig. 10:**
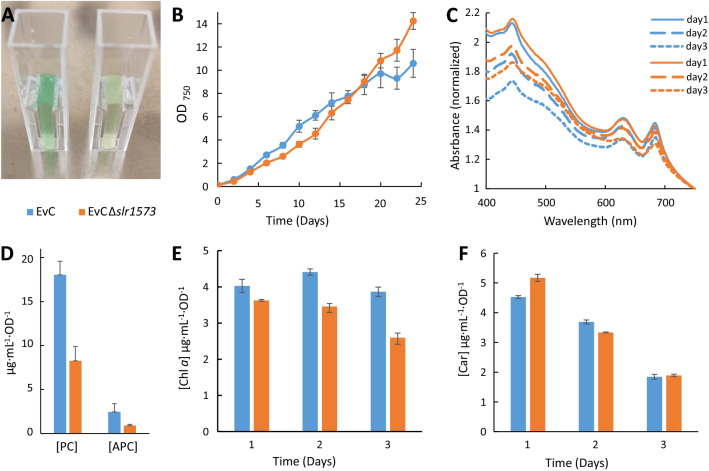
Comparison of strains EvC to EvC*Δslr1573*. **A** Picture of both cultures at OD_750_ approx. 0.4, left—EvC, right—EvC*Δslr1573*; **B** growth curves during 24 days of cultivation; **C** whole cell absorbance spectra normalized at 750 nm; **D** phycobiliprotein concentration normalized per OD_750_ taken at day 3; **E** Chlorophyll *a* content normalized per OD_750_; **F** total carotenoid content normalized per OD_750_

We also observed a notable difference in phenotype of Δ*slr1573* strains compared to the strains with an intact *slr1573*. The color of the Δ*slr1573* strains was comparably more yellowish with less green (Fig. 10A). To address this phenotype difference, we performed a prolonged growth experiment for both control strains as well as pigments quantification. The growth of EvCΔ*slr1573* was considerably slower than that of EvC in the first half of the linear phase (Fig. 10B).Whole cell absorbance spectra was measured in the beginning of the growth experiment, before both cultures reached an OD_750_ of 1, as for older cultures a shading effect may contribute to the difference in pigment content. The recorded spectra showed distinct absorbance patterns for the two strains (Fig. 10C). Pigment content quantification was also carried out at the same time points as the whole spectra. The results (Fig. 10D–F) showed that EvCΔ*slr1573* strain has markedly less phycobiliproteins as well as less chlorophyll *a* per cell, while carotenoid content was higher, although only in the beginning of the experiment. It is not clear what mechanisms cause these effects, whether it is a direct effect on the pigment synthesis or regulation in the cell, or a secondary effect due to stress conditions caused by the deletion of *slr1573*.

All together, the abovementioned data indicate that deletion of the *slr1573* laccase in *Synechocystis* has a clear impact on the cells, which has not been previously reported. Although laccases and laccases-like enzymes are very abundant in nature and have many diverse functions, their role in bacteria, and especially in cyanobacteria has not been investigated extensively [44].

### Production of *t*CA under small-scale high density cultivation (HDC) conditions

The specific production titers of *t*CA and *p*Cou obtained in the above described experiments are lower compared to those that were reported previously, although we find it difficult to compare them due to different normalization and growth conditions used. The levels of *t*CA and *p*Cou produced in cyanobacteria reported to date are: 114 mg L^−1^ (267 ± 31 mg gDW^−1^) of *t*CA and 207 mg L^−1^ (470 ± 70 mg gDW^−1^) of *p*Cou obtained after growth in Multi-cultivator MC 1000OD, under 100 µmol photons m^−2^ s^−1^ and with air enriched with 3% (v/v) CO_2_ [25]; 197 mg L^−1^ of *p*Cou after 7 days of cultivation under 50 µmol photons m^−2^ s^−1^ [27]; 82.6 mg L^−1^ of *p*Cou after 7 day cultivation under 50 µmol photons m^−2^ s^−1^ with 5 mM glucose supplementation in the media [26].

**Fig. 11:**
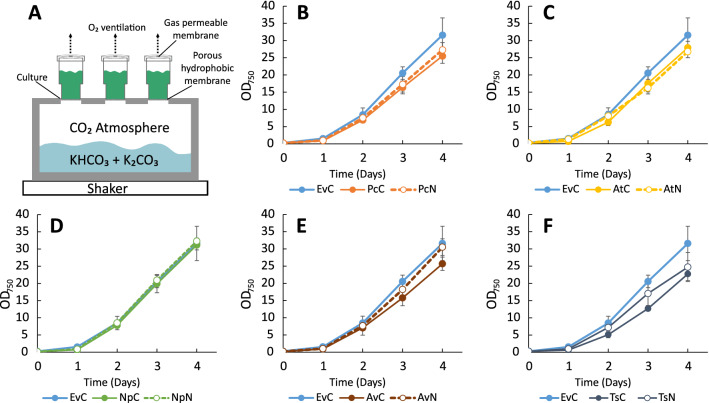
Growth of *Synechocystis* engineered strains during high-density cultivation for 4 days. **A** schematic representation of the CellDeg HDC 6.10B system. The cultivation bath is filled with 3 M carbonate buffer and constantly creates a CO_2_ rich atmosphere with diffuses through a porous hydrophobic membrane to the culture medium. Oxygen ventilation of growing cultures is provided by gas permeable membrane outlet on the lid of cultivation vessel. **B** growth of strains EvC, PcC, PcN; **C** strains EvC AtC, AtN; **D** strains EvC, NpC, NpN; **E** strains EvC, AvC, AvN; **F** strains EvC, TsC TsN. For each strain at least two independent cultivations experiments with biological triplicates was carried out. Error bars represent standard deviation

Therefore, we intended to test whether we can reach high volumetric titer of molecules of interest using improved growth conditions, such as small-scale high-density cultivation. We used a cultivation system from CellDEG (www.celldeg.com) (Fig. 11A), which has been used to overcome crucial high-density cultivation limitations such as CO_2_ supply and uneven light distribution in dense cultures for successful production of cyanophycin and terpenoids reaching ultra-high cell densities in a short period of time [31–33].

**Fig. 12:**
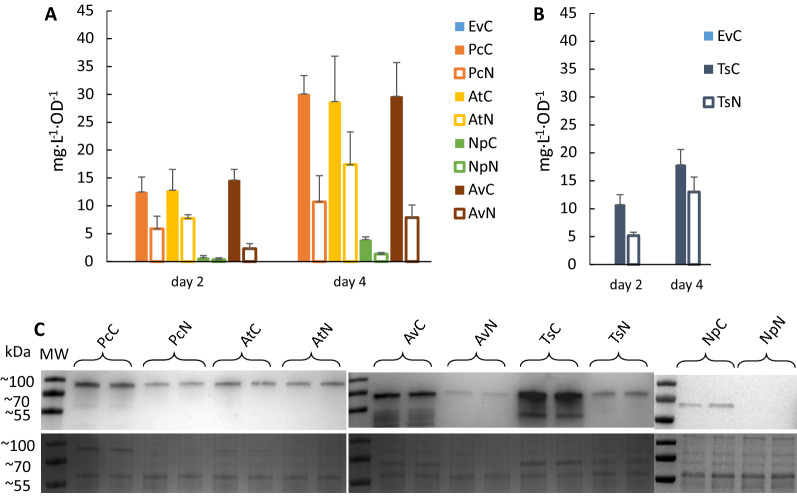
*t*CA and *p*Cou production and Western Blot of engineered *Synechocystis* strains during high-density cultivation experiments. **A** *t*CA production in engineered *Synechocystis* strains EvC, PcC, PcN, AtC, AtN, NpC, NpN, AvC, AvN. **B** *p*Cou production in strains TsC and TsN. Samples of growth medium were taken on day two and four. The values are the means of three biological replicates and two technical replicates, error bars represent the standard deviation. **C** WesternBlot and SDS-PAGE of extracts from engineered *Synechocystis* strains (EvC, PcC, PcN, AtC, AtN, NpC, NpN, AvC, AvN, TsC and TsN) analyzed for presence of PAL proteins taken on day 4 of HDC experiment. Upper panel displays the Western Blot using anti-Strep antibody; bottom panel shows the SDS-PAGE loaded with 3 µg of soluble fraction from protein crude extract of the different strains

For HDC experiments, we used the production strains based on the pEEK expression vectors. The *Ts*-H87L strains were not included since the productivity of *t*CA was the lowest for those. The cultures of engineered *Synechocystis* were grown for four days, and samples for LC–MS were taken on days two and four. Figure 11B–F displays the growth curves, where the culture density after four days of cultivation reached values in the range of OD_750_ 25–32. Similarly to what was observed before (Fig. 4), the growth pattern of engineered strains that had the least productivity showed similar growth to the EvC (NpC and NpN) whereas the other reached lower OD compared to EvC. The productivity results (Fig. 12, Table 3) showed that the specific *t*CA and *p*Cou titers at the end of experiment are lower than during the Erlenmeyer-flask cultivation for each corresponding strain. The relative expression of PALs (Fig. 12C) showed considerably higher protein levels in strains AvC and PcC than AtC, which presumably led to higher *t*CA titer per cell than in the AtC strain, unlike the results from standard growth conditions where strain AtC had the highest production.

**Table 3:** *t*CA and *p*Cou production by engineered *Synechocystis* strains after four days of high-density cultivation

Strain	mg L^−1^ OD_750_^−1^	mg L^−1^
***t*CA production**		
EvC	N/D	
PcC	30.1 ± 3.3	746.6 ± 51.2
PcN	13.1 ± 2.4	369.9 ± 83.8
AtC	28.7 ± 8.2	771.3 ± 233.9
AtN	17.4 ± 5.9	539.3 ± 134.1
NpC	3.9 ± 0.5	123.4 ± 19.8
NpN	1.3 ± 0.3	43 ± 9
AvC	29.7 ± 6.1	797.8 ± 153.3
AvN	7.9 ± 2.3	238.0 ± 62.5
***p*Cou production**		
TsC	17.9 ± 2.7	411.6 ± 94.9
TsN	13 ± 2.7	313 ± 35.3

Data correspond to the panels A and B in Fig. 12

N/D, none detected

Although the specific production titer was compromised for HDC, as it was observed previously [32], the high biomass accumulation rate resulted in high total product yield of 797.8 ± 153.3 mg L^−1^ and 411.6 ± 94.9 mg L^−1^ of *t*CA and *p*Cou respectively (Table 3), which is the highest reported in cyanobacteria so far. In summary, this experiment shows a great potential for obtaining high volumetric concentrations of a product of interest via optimized growth conditions, allowing the cultures to reach very high biomass concentration within a short time period.

## Conclusions

In this study we aimed to express several *pal* genes in *Synechocystis* and subsequently evaluate the production of *t*CA by engineered strains in order to select the best performing candidate. Out of five selected *pal* genes from different organisms, PAL from *Treponema socranskii* turned out to be a TAL, but a single amino acid substitution in substrate selectivity switch position was sufficient to change the substrate preference to Phe. Comparative growth and production experiments showed that the best performing stain was AtC with the specific production of 40.2 ± 5.6 mg L^−1^ OD_750_^−1^ in six days. Further improvement of productivity by the increase of expression levels of PAL proteins did not result in higher specific production titers, although several strains showed nearly similar productivity due to similar relative PALs expression. Knock-out of the *slr1573* laccase gene, which has been suggested to be active in the degradation of synthetized *t*CA and *p*Cou, did not result in any improvement of productivity. However, we could observe strong phenotype of slower growth and lower pigments content for the strains lacking the *slr1573* laccase. The application of a high-density cultivation platform for growth of the engineered strains resulted in a remarkably high *t*CA volumetric titers of 797.8 ± 153.3 mg L^−1^ by strain AvC, consistent with the central role of Phe in the cell metabolism which ensures a high flux of precursors for its synthesis, and subsequently for the production of *t*CA. In summary, this work contributes to the development of cyanobacteria as cell factories for sustainable conversion of CO_2_ into phenylpropanoids.

## Methods

### Bacterial strains and growth conditions

*Escherichia coli* DH5 αZ1 (Invitrogen) was used for subcloning and conjugation. *Escherichia coli* cells were grown in LB medium at 37 °C and supplemented with appropriate antibiotics to the final concentrations in the medium: 50 μg ml^−1^ kanamycin (Km) or/and 20 μg ml^−1^ chloramphenicol (Cm) (Sigma, Merk).

*Synechocystis* sp. PCC 6803, a unicellular glucose-tolerant strain was used in this study. Cultures were grown in BG11 medium [45] with respective antibiotics Km 25 μg ml^−1^ and/or Cm 20 μg ml^−1^ at 30 °C under constant light. The optical density of *Synechocystis* cultures at 750 nm was measured using a Varian Cary 50 BIO spectrophotometer, and an OD_750_ value of 1 corresponds to approximately 10^8^ cells.

### Construction of plasmids for gene expression

The broad host range self-replicative vectors pEEK and pEEK3 [37] (also Englund E., not published) which are based on the vector pPMQAK1 [46] were used in this study as shuttle vectors (Fig. 2). The pEEK vector carries a Strep-tag with glycine-serine linker at the C-terminus position and the counter-selection marker *ccdB* between XbaI and BglII site. The expression is driven by a strong constitutive promotor P*trc2O* and the translation is initiated from the synthetic ribosomal binding site RBS* [47]. In order to evaluate an optimal condition of PAL proteins activity, another vector pEEKN was constructed, which in contrast to pEEK, can be used to place a Strep-tag at the N-terminus, and the *ccdB* sequence is flanked by BamHI and SpeI. Vector pEEK3 is different from pEEK and pEEKN by containing the strong constitutive promotor P*trcCore* and a RiboJ element to give strong reliable expression [42]. Based on pEEK3 another vector pEEK3C was constructed, where downstream of RiboJ sequence strong bacterial RBS [48] is placed, followed by XbaI and BglII restriction sites flanking *ccdB* sequence and a glycine-serine linker with Strep-tag placed at the C-terminus of the expressed protein.

*pal* from *Nostoc punctiforme* ATCC 29133/PCC 73102 (UniProtKB—B2J528) was amplified using genomic DNA as a template. The rest of the *pal* genes used in this study, from *Arabidopsis thaliana* (UniProtKB—P45724), *Petroselinum crispum* (UniProtKB—P24481), *Anabaena variabilis* ATCC 29413/PCC 7937 (*Trichormus variabilis*) (UniProtKB—Q3M5Z3) and *Treponema socranskii subsp. paredis ATCC 35535* (UniProtKB—S3JNX8), were codon optimized using Gene Designer (DNA2.0) software for heterologous expression in *Synechocystis* and synthesized by GenScript.

Genes were inserted into plasmids using SpeI and BamHI (pEEK), BamHI, and SpeI (pEEK3) restriction sites. For insertion into pEEK3C vector, genes were likewise digested with SpeI and BamHI, but ligated into XbaI and BglII sites on the plasmid backbone, resulting in creation of the TCTAGT and GGATCT scars, respectively. For insertion into pEEKN, BglII and SpeI restriction sites were used for *Pc*, *At* and *Av pals,* BamHI and SpeI for *Np pal,* BglII and PstI for *Ts tal*.

Site directed mutagenesis of *Ts tal* sequence was performed with *Ts tal* cloned into pEEK and pEEKN as template using 5′phosphorelated primers carrying overhang for substitution of two nucleotides at position Histidine 87 to create Leucine 87. The linear DNA fragment was then ligated and transformed in *E. coli*.

The laccase oxidase encoded by open reading frame *slr1573* in *Synechocystis* [26] was knocked out by replacing the gene with a Cm resistance cassette. For this, 1 kb flanking regions upstream and downstream of *slr1573* gene were amplified from *Synechocystis* genomic DNA and cloned into integrative vector pEERM3 [39] creating the plasmid p1573 (Fig. 2F).

### Conjugation and transformation of *Synechocystis*

For conjugation, overnight cultures of *E. coli* cargo cells and *E. coli* HB101 helper cells with pRL443-Amp^R^ plasmid were centrifuged at 5000 rpm for 5 min and resuspended in fresh LB medium without antibiotics. A mixture of cargo cells (1 ml), helper cells (1 ml) and wild-type *Synechocystis* PCC 6803 (200 µl) was incubated under 100 µmol photons m^−2^ s^−1^ at 30 °C for 2 h. The mixture was then spread on a filter on a BG11 agar plate without antibiotics for another 24 h of incubation. For colony selection, the filters were changed onto new BG11 agar plates with 50 µg ml^−1^ Km. Colonies appeared after 1–2 weeks and were screened by PCR using gene specific primers and DreamTaq DNA polymerase (Thermo Fisher Scientific). Positive colonies were inoculated in fresh liquid BG11 medium with 25 µg ml^−1^ Km.

For transformation, *Synechocystis* engineered strains possessing already the Km resistance, were transformed with p1573 as described previously [46]. Colonies that appeared after 10–14 days were analyzed using PCR and restreaked on plates repeatedly until full segregation was reached.

### Determination of *t*CA and *p*Cou by LC–MS

Determination of *t*CA and *p*Cou in the growth media was performed by LC–MS. For this 1 ml of supernatant was taken from *Synechocystis* cultures at certain days of experiment, samples were filtered through 0.2 μm pore PTFE filters (Fisherbrand) and subjected to HPLC analysis. Samples were stored at − 20 °C if not analyzed the same day.

For qualitative analysis of *E. coli* strains for the presence of corresponding compounds, 3 ml of liquid cultures were inoculated from overnight seed cultures, grown until OD_600_ ~ 1 and induced with 1 mM of IPTG (Sigma, Merk). After induction, cells were grown for another 3 h and 1 ml of supernatant was collected. The samples were treated analogously as of *Synechocystis* cultures.

HPLC–MS analysis was performed using an Agilent 1290 Infinity II HPLC system equipped with a 1290 Infinity II High Speed pump and a 1260 II Infinity DAD HS UV–vis detector, using an InfinityLab POROSHELL SB-120 C18 column with dimensions of 50 mm × 2.1 mm and 2.7 μm particle size. The HPLC was coupled to an InfinityLab LC/MSD equipped with an ESI ionization source. LC separation was performed using a water (A, 0.1% formic acid) and acetonitrile (B) eluent system using the method: 0–1 min 10% B; 1–10 min 10 → 90% B; 10–11 min 90% B; 11–11.1 min 90–10% B, 11.1–12 min 10% B; at the flow rate of 0.3 ml/min. The quantification of *t*CA and *p*Cou in *Synechocystis* cultures was based on a linear calibration curve from standards measured in technical triplicates. Standards for *t*CA and *p*Cou (Sigma, Merk) were prepared in BG11 medium in the range 1–100 μg·ml^−1^ and filtered before analysis.

### Western blot (WB) analysis

Proteins from *Synechocystis* cells were extracted on the last day of the experiment as described by Ivleva and Golden 2007 [49], and the soluble fraction from the crude cell extracts was used further for the analysis. Protein quantification was performed with DC protein assay (Bio-Rad), using albumin from bovine serum (Sigma) as a standard. Soluble proteins were separated by SDS-PAGE, using Mini-PROTEAN TGX™ gels (Bio-Rad), and transferred to PVDF membrane (Bio-Rad.) Immunoblot was performed according to standard techniques using Anti-Strep-tag II (Abcam) for the detection of Strep-tagged proteins.

### Pigments quantification

To determine pigment content in strains EvC and EvCΔ*slr1573*, the pre-cultures inoculated from cryostocks were grown in triplicates under 45 µmol photons m^−2^ s^−1^ for several days. Then, cultures were re-inoculated with fresh media to the starting OD_750_ ~ 0.1. Samples for chlorophyll *a* and carotenoid quantification (1 ml) were collected in triplicates on the day after starting the experiment (day 1) and the following two days (day 2 and day 3), until the cultures reached OD_750_ ~ 1.The cells were then centrifuged for 2 min at 15,000×*g*, the supernatant was removed and 1 ml of methanol 100% (Alpha Aesar) was added. Samples were homogenized and incubated in dark at 4 °C for at least 30 min. After incubation, the samples were centrifuged for 10 min at 15,000×*g* at 4 °C, and the supernatant was then used to measure absorbance at 470 nm, 665 nm and 720 nm in a Varian Cary 50 BIO spectrophotometer, using methanol as blank. Concentration of chlorophyll *a* and carotenoids were calculated according to Ritchie et al. [50] and Wellburn et al. [51] respectively.

For phycobiliproteins determination, culture samples (2 ml) were collected at day 3 in triplicates and centrifuged at 15,000×*g* for 2 min. The supernatant was discarded and acid-washed glass beads (425–600 μm diameter, Sigma-Aldrich) were added to the sample together with 200 µl of PBS solution. The cells were disrupted using the Precellys-24 Beadbeater (Bertin Instruments) using program 3 × 30 s. Then 800 µl of PBS was added to the sample, the mixture was mixed vigorously and incubated for at least 1 h. After, the samples were centrifuged for 5 min, the supernatant transferred to a new tube and centrifuged at maximum speed for 30 min at 4 °C. The absorbance values at 652 nm and 615 nm were measured and concentration of allophycocyanin (APC) and phycocyanin (PC) was determined according to Bennet and Bogorad [52].

### High-density cultivation

For the small-scale high-density cultivation an HDC 6.10 starter kit (CellDEG, Germany) was used. The kit consist of 10 ml cultivation vessels with porous hydrophobic membrane at the bottom and a buffer reservoir which was filled with 200 ml of a 3 M KHCO_3_/3 M K_2_CO_3_ (9:1 ratio) solution to provide 90 mbar partial pressure of CO_2_ (reference T = 20 °C, according to the manufacturer’s recommendation). The nutrient-enriched media was prepared as described in Lippi et al. [33] with slight modifications: as an iron source ammonium iron (III) citrate was used and sodium nitrate as a sole nitrate source.

The cultivation was carried out in a “Versatile Environmental Test Chamber” (Sanyo) w/o humidifier at 30 °C under multidirectional illumination with fluorescent white light with increasing light intensities: 250 μE m^−2^ s^−1^ (0–24 h), 490 μE m^−2^ s^−1^ (24–48 h), 750 μE m^−2^ s^−1^ (48–96 h). The CellDEG system was shaking constantly at 320 rpm (IKA KS 130 basic orbital shaker ø = 4 mm).

## Supplementary Information

**Additional file 1: Figure S1.** LC–MS profile of *E. coli* culture medium at 275 nm wavelength. Retention time of 4.64 and 6.39 min corresponds to the *p*Cou and *t*CA standards correspondingly. **A** profile from strain harboring pEEK* plasmid; **B** profile from strain harboring *Ts tal* in pEEK vector. **C** profile from strain harboring *Ts*-H87L *pal* in pEEK vector. **Figure S2.** Mass spectrum of the peak at 5.669 min in culture growth medium of *EvCΔslr1573* strain. **A** positive ionization; **B** negative ionization.

## Data Availability

The datasets used and/or analysed during the current study are available from the corresponding author on reasonable request.

## Abbreviations

AAA: Aromatic amino acids
APC: Allophycocyanin
*At*: *Arabidopsis thaliana*
*Av*: *Anabaena variabilis*
C4H: Cinnamate-4-hydroxylase
CBB cycle: Calvin-Benson-Bassham cycle
Cm: Chloramphenicol
DAHP: 3-Deoxy-d-arabinoheptulosonate 7-phosphate synthase
*E.** coli*: *Escherichia coli*
E4P: Erythrose-4-phosphate
EPSP: 5-Enolpyruvylshikimate-3-phosphate synthase
HAL: Histidine ammonia lyase
HDC: High-density cultivation
Km: Kanamycin
MIO: 3,5-Dihydro-5-methylidine-4H-imidazol-4-one
*Np*: *Nostoc punctiforme*
PAL: Phenylalanine ammonia lyase
*Pc*: *Petroselinum crispum*
PC: Phycocyanin
*p*Cou: *p-*Coumaric acid
PEP: Phosphoenolpyruvate
Phe: Phenylalanine
PQ: Plastoquinone
*Synechocystis*: *Synechocystis* sp. PCC 6803
TAL: Tyrosine ammonia lyase
*t*CA: *trans*-Cinnamic acid
Trp: Tryptophan
*Ts*: *Treponema socranskii subsp. paredis* ATCC 35535
Tyr: Tyrosine
WB: Western blot
WT: Wild type
