# Supplementary material for: Expression of phenylalanine ammonia lyases in Synechocystis sp. PCC 6803 and subsequent improvements of sustainable production of phenylpropanoids
Source: Microb Cell Fact. 2022 Jan 10;21:8. doi: 10.1186/s12934-021-01735-8 (PMC8750797; doi:10.1186/s12934-021-01735-8)
Supplement: Supplementary file 1 — Additional file 1: Figure S1. LC–MS profile of E. coli culture medium at 275 nm wavelength. Retention time of 4.64 and 6.39 min corresponds to the pCou and tCA standards correspondingly. A profile from strain harboring pEEK* plasmid; B profile from strain harboring Ts tal in pEEK vector. C profile from strain harboring Ts-H87L pal in pEEK vector. Figure S2. Mass spectrum of the peak at 5.669 min in culture growth medium of EvCΔslr1573 strain. A positive ionization; B negative ionization. [file 12934_2021_1735_MOESM1_ESM.docx]

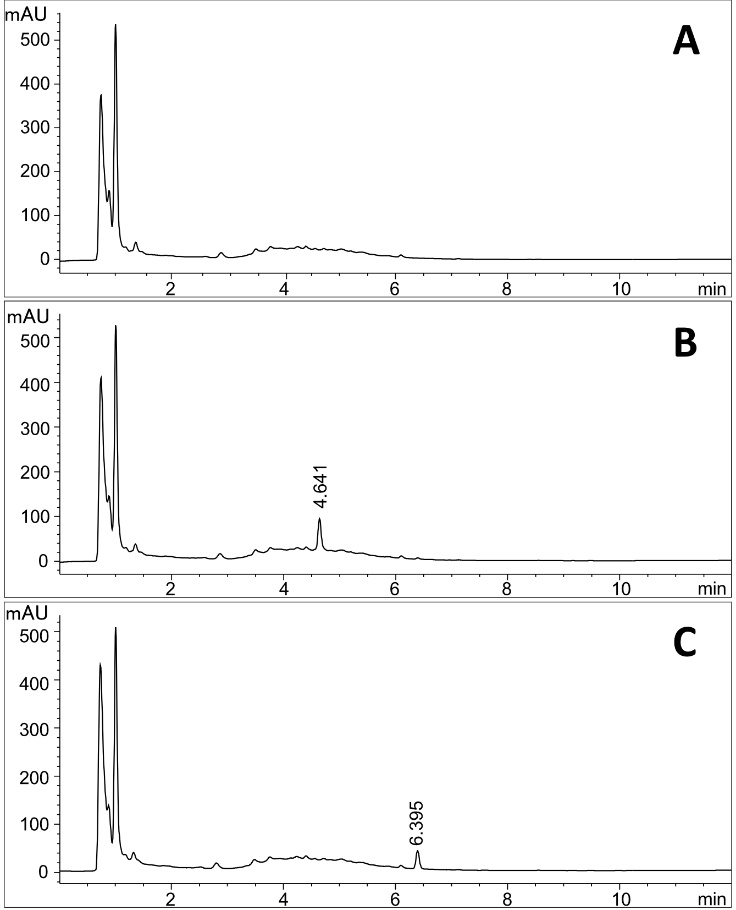


**Figure S1. LC-MS profile of *E.coli* culture medium at 275 nm wavelength.** A retention time of 4.64 and 6.39 minutes corresponds to the *p*Cou and *t*CA standards, respectively. **A** profile from strain harboring pEEK* plasmid; **B** profile from strain harboring *Ts* *tal* in pEEK vector. **C** profile from strain harboring *Ts*-H87L *pal* in pEEK vector.


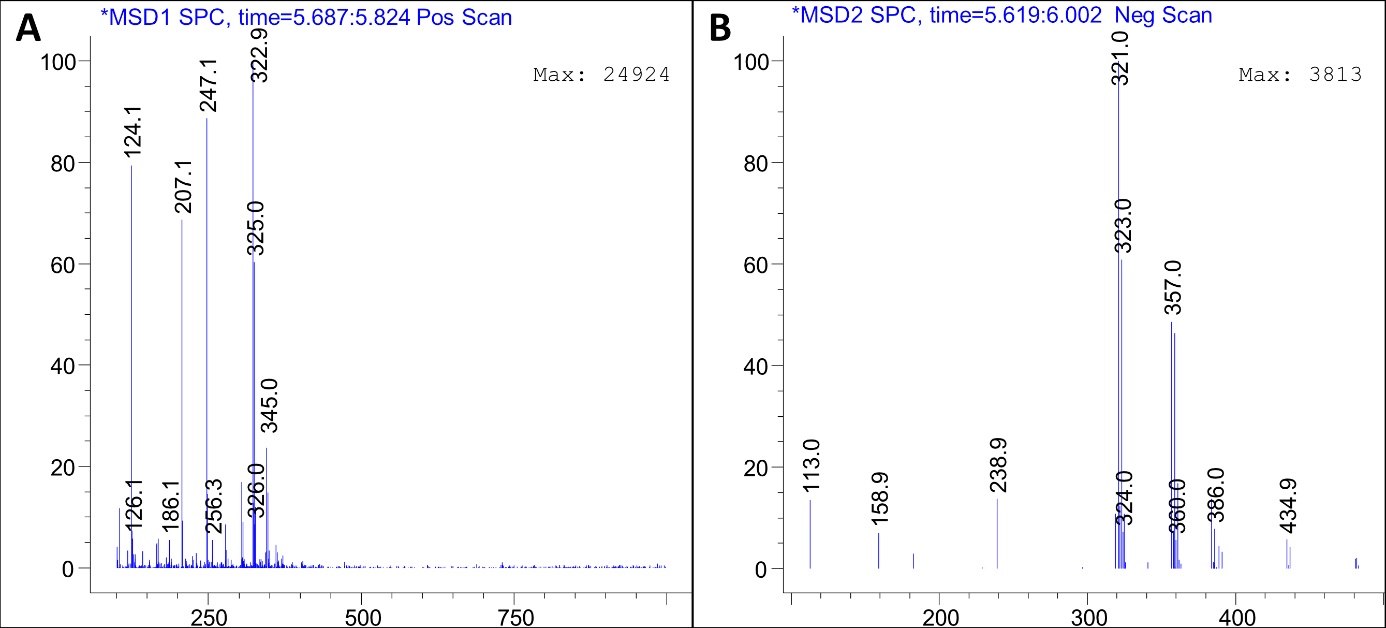


**Figure S2. Mass spectrum of the peak at 5.669 min in culture growth medium of EvC*Δslr1573* strain. A** positive ionization; **B** negative ionization.
